# Porphyrin-Grafted Poly(ethylene terephthalate) as a Reusable and Highly Selective Colorimetric Probe for Mercuric Ion Contaminants in Aqueous Samples

**DOI:** 10.1021/acsami.4c03846

**Published:** 2024-07-22

**Authors:** Eduardo C. Atayde, Yasumasa Takenaka, Hideki Abe, Ming-Rou Wu, Kevin C.-W. Wu

**Affiliations:** † Molecular Science and Technology Program, Taiwan International Graduate Program, Academia Sinica, No. 128, Sec. 2, Academia Road, Taipei 11529, Taiwan; ‡ Department of Chemistry, National Tsing Hua University, No. 101 Sec. 2, Kuang-Fu Road, Hsinchu 30013, Taiwan; § Department of Chemical Engineering, National Taiwan University, No. 1, Sec. 4, Roosevelt Road, Taipei 10617, Taiwan; ∥ Bioplastic Research Team, RIKEN Center for Sustainable Resource Science, 2-1 Hirosawa, Wako, Saitama 351-0198, Japan; ⊥ Department of Chemical Engineering and Materials Science, Yuan Ze University, No. 135, Yuandong Rd., Zhongli District, Taoyuan 32003, Taiwan; # Department of Chemical Engineering, Chung Yuan Christian University, No. 200, Zhongbei Rd., Zhongli Dist., Taoyuan 320, Taiwan

**Keywords:** colorimetric probe, mercuric ion, oxazoline, PET, porphyrin

## Abstract

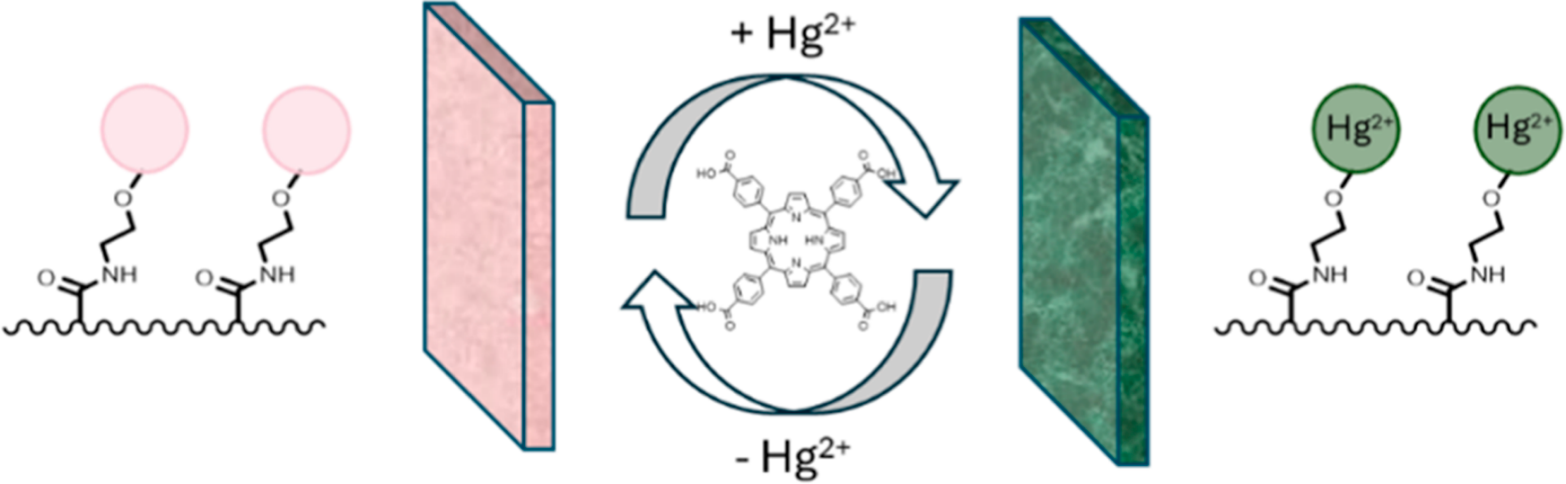

Heavy metals are the most hazardous water pollutants, with severe health and environmental consequences. Among these, mercuric (Hg^2+^) ions are known to cause detrimental health issues in both humans and aquatic life. Due to this, several analytical techniques have been devised to detect and quantify the amount of this ion. However, most of these require advanced instrumentation, prolonged analysis time, and sample preparation. In this study, a low-cost and highly reusable colorimetric probe was developed by grafting porphyrin to poly(ethylene terephthalate) sheets using an oxazoline polymer as covalent adhesive. Upon exposure to trace amounts of Hg^2+^ in solution, the fabricated material visually transitioned from faint brownish pink to green by the complexation mechanism. Additionally, the transparency of this probe allowed the quantitative spectrophotometric determination of the Hg^2+^ concentration in aqueous samples. It was also shown that the material is highly stable, which can be reused for more than 50 times without significant decline in its performance, hence, making it suitable for the onsite monitoring of mercuric ion contamination in different bodies of water.

## 1 Introduction

The increase in global population, industrialization, and waste accumulation has led to the perennial problem of pollution that the world faces today. Among these issues is water pollution, which poses a serious threat not only to marine biodiversity but also to human health. A report published by the United Nations Environment Program revealed that among the 75,000 bodies of water in 89 countries, more than 40% were found to be severely polluted.^1^ A major contributor to water pollution is plastic, which constitutes 80% of all marine debris found from surface waters to deep-sea sediments, with an estimated 14 million tons of plastic polluting the oceans annually.^2^

Another critical issue related to water pollution is the contamination of water resources with heavy metals. Although not as widespread as plastic accumulation, heavy metals are known to be the most hazardous pollutants, with severe health and environmental consequences even at low concentrations.^3−5^ For instance, human exposure to mercuric ions can cause grave health issues primarily affecting the gastrointestinal tract and the kidneys. Mercuric ions were also found to accumulate in the brain via one or more amino acid transporters and may deposit periportally in the liver.^6,7^ Due to these risks and the tendency of mercury to bioaccumulate,^4^ several protocols have been developed to accurately determine Hg^2+^ amounts in water samples. However, these methods often require complex instrumentation, lengthy analysis times, and tedious sample preparation.^8−12^ It is, therefore, imperative to explore low-cost, straightforward, and reliable alternatives for the rapid detection of mercury contamination in water resources that can also be employed for onsite monitoring.

Recent advancements have led to the creation of several Hg^2+^ sensors, utilizing probes like metal oxo-clusters,^13,14^ Au/Ag nanoparticles,^15,16^ quantum dots,^17^ polymers,^18^ and metal–organic frameworks,^19^ with quantification methods that are generally based on the material’s fluorescence, UV–vis absorbance, electrochemistry, or surface plasmon resonance.^20,21^ Only a small percentage of the published papers, however, reported on sensors that can also be amenable to actual onsite monitoring by utilizing a perceptible change in the material’s property such as its color.^22−24^

Porphyrin, a naturally occurring macrocyclic ring found in blood heme, can interact with metal ions through complexation with its vacant metal center. It is a highly absorbing molecule whose absorbance profile changes with the metal ion bound to its macrocyclic pyrrole center.^25^ Its extensive conjugation allows it to manifest different colors that are noticeable to the naked eye when exposed to variable conditions. These remarkable properties render porphyrin a suitable probe in the development of colorimetric sensors for detecting Hg^2+^ and other heavy metals.^26^ While there have been numerous reports on colorimetric porphyrin-based sensors for Hg^2+^, most of them are homogeneous and thus limited to single time use.^27,28^ Consequently, attempts were made to produce reusable porphyrin probes deposited on solid substrates. However, most methodologies require stringent synthetic and fabrication procedures to create a functional material.^29−31^ For example, Zhang and co-workers developed a reliable and portable colorimetric Hg^2+^ probe based on the porphyrin-functionalized polyacrylonitrile fiber, which can be directly used for onsite analysis as it can visibly change color upon exposure to Hg^2+^-spiked aqueous samples. Despite its effectiveness, its fabrication required complicated synthetic steps involving porphyrin functionalization and its chemical attachment to the nanofiber.^32^ Another study utilized a cationic porphyrin derivative, which was self-assembled on a glass substrate and was reported to demonstrate a very low limit of detection for Hg^2+^ sensing via fluorescence measurements. The main drawback, though, is the seven-step complicated synthesis of the functionalized porphyrin and the need for sophisticated instrumentation, which limits its practicality for onsite applications.^30^ Moreover, Ermakova and co-workers deposited different porphyrin derivatives on polyvinyl chloride (PVC), which provided a stable solid support. The fabricated material was proven to be reusable and capable of the onsite qualitative detection of Hg^2+^ by monitoring any color change in the film. The probe’s response could also be further quantified by utilizing both fluorescence and UV–vis spectroscopy, achieving a low LOD of 2 ppb. The main challenge, however, is the difficult muti-step synthesis of the porphyrin derivatives and the need for an advance instrument in both the fabrication of the Langmuir monolayers and their deposition on PVC substrates.^33^ Recently, Caroleo et al. reported the use of a cheap Colour Catcher sheet as a low-cost porphyrin support for mercury (II) ion detection. Although their sensor can detect trace amounts of the contaminant, assessment of the material’s reusability was not conducted.^34^ Intriguingly, there have been no reports on the use of poly(ethylene terephthalate) (PET) as a substrate in the development of porphyrin-based sensors. PET’s flexibility, high stability, lightweight, and transparency make it an excellent substrate for highly reusable and stable colorimetric sensing material.^35^ Moreover, utilizing PET offers an additional strategy to upscale plastic wastes, thereby contributing to the reduction of plastic pollution.

In this study, we report for the first time a low-cost, reliable, and reusable porphyrin-grafted PET probe that can be fabricated through a facile approach and can selectively detect Hg^2+^ ions even at very low concentrations. Its ability to exhibit an observable color change upon exposure to Hg^2+^ renders it suitable for the onsite qualitative determination of mercury in various water sources. Moreover, the transparency of the material allows for further quantitative analysis using ultraviolet–visible spectrophotometry.

## 2 Methods

### 2.1 Measurements

The films were analyzed by Fourier transform infrared (FT-IR) spectroscopy (Shimadzu IRPresitge-21) and microscopy (Olympus BX53) to monitor the different stages of material deposition on the PET substrates. The thermal stability of the fabricated films was examined by using a thermogravimetric analyzer (Exstar TG/DTA7200). UV–vis–NIR spectrophotometry (Shimadzu UV-3600) was utilized for the spectrochemical characterization of the grafted porphyrin and the quantitative analysis of the chelated Hg^2+^. The morphology and thickness of the material were analyzed by field-emission scanning electron microscopy (JEOL JSM-7900F) operating at an accelerating voltage of 10 kV. Low-resolution and high-resolution X-ray photoelectron spectroscopy (XPS) analyses (ULVAC PHI 5000 Versa Probe) were also performed to provide insights into the complex formation between Hg^2+^ and the probe. Inductively coupled plasma–mass (ICP–MS) (Thermo Fisher Scientific) was also employed to indirectly quantify the amount of porphyrin moieties that are present on the surface of the material.

### 2.2 Surface Modification to Produce −COOH-Enriched PET^36,37^

PET films (2 cm × 2 cm, 100 μm thickness) were initially etched by immersion and shaking in 2 M NaOH solution for 6 h at 60 °C. The films were then washed with deionized water and subsequently shaken in concentrated acetic acid for 1 h at 60 °C. The resulting films were again washed with deionized water and dried in an oven. This etching process afforded the translucent PET films. Two separate methods were employed for the oxidation of the films. UV-induced oxidation: the etched films were immersed in 600 mM H_2_O_2_ at pH = 3 (HCl) and were placed in a black box and exposed to UV irradiation at 254 nm using a mercury lamp with an output of 190 W for 1.5 and 3 h at room temperature. KMnO_4_oxidation: the films were submerged in 5% KMnO_4_ in 1.2 N H_2_SO_4_ solution and were then shaken at varying temperatures and periods. Then, the films were washed sequentially with 9 M HCl, 4.5 M HCl, and 50:50 ethanol/water solution. The resulting films were then dried in an oven set at 60 °C.

### 2.3 Analysis of the Amount of Carboxylic Acids on the PET Surface^38^

The oxidized PET films were shaken in 0.5 mM toluidine blue (TBO) in deionized water (pH 10) for 2 h at room temperature. The films were then washed with deionized water at pH 10 to remove the unreacted TBO. After this, the complexed TBO on the PET surface was then desorbed by immersion and agitation of the films in 50% acetic acid for 6 h. The absorbance of the TBO dissolved in acetic acid was then analyzed using UV–vis spectrophotometry at 633 nm, and its corresponding concentration was computed using a calibration curve generated from the standard solutions of TBO in 50% acetic acid.

### 2.4 Deposition of the Oxazoline-Functionalized Polymer on the COOH-Enriched PET Surface

A 400 μL solution of the oxazoline-functionalized polymer (EPOCROS WS-700, Nippon Shokubai Co. Ltd.) was spin coated on the oxidized PET film at 3000 rpm for 70 s. Thereafter, the dried material was baked at 110 °C for 1 h.

### 2.5 Grafting of Tetrakis(4-Carboxyphenyl)porphyrin on the Oxazoline-Functionalized PET

The oxazoline-functionalized PET film was submerged in 2 mL of 8 mg/mL solution of tetrakis(4-carboxyphenyl)porphyrin (TCPP) in super dehydrated dimethylformamide (DMF). The mixture was subsequently heated at 110 °C for different periods. The grafted film was then washed sequentially with DMF and acetone. After this, the film was dried at 60 °C in an oven, affording a pale brownish pink film.

### 2.6 Hg^2+^ Detection Using the TCPP-Grafted PET Film

The porphyrin-grafted PET film was shaken in 10 mL of an aqueous sample containing Hg^2+^ ions until a noticeable transition to a green color was observed. The duration of shaking depended on the amount of ions present in the water samples. To reuse the film, it was agitated in 0.1 M HCl for 30 s and washed with deionized water until the film reverted to its initial color. To quantify the amount of Hg^2+^, the green film was subjected to UV–vis analysis.

## 3 Results and Discussion

### 3.1 Surface Modification to Produce −COOH-Enriched PET

**Scheme 1:**
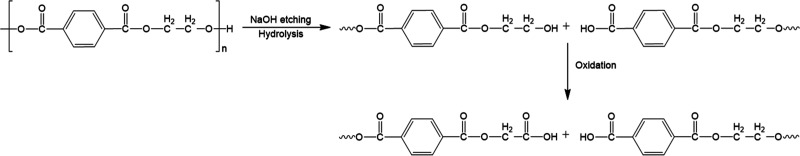
Surface Functionalization of PET Substrates by Etching and Oxidation

To ensure adequate anchoring points for porphyrin grafting, it was necessary to enrich the PET with carboxylic acid (−COOH) groups, as shown in Scheme 1. This was carried out by first etching the film with NaOH, which hydrolyzed the ester groups to generate both hydroxyl (−OH) and carboxyl moieties (−COOH) on the surface of the film.^36^ Subsequent oxidation by either H_2_O_2_/UV or KMnO_4_ further converted the available hydroxyl groups to carboxylic acids.

**Figure 1:**
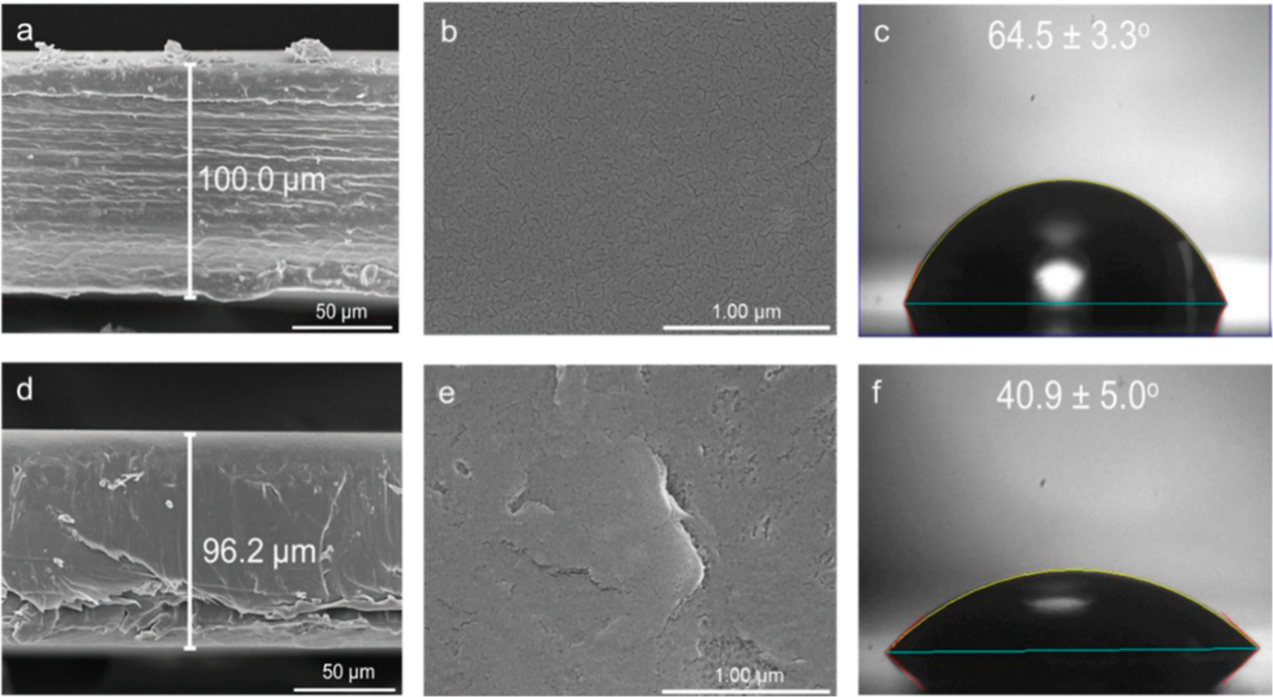
(a,d) SEM images of the PET thickness, (b,e) surface, and (c,f) water contact angle measurements before and after surface modification.

From the SEM images in Figure 1, it is evident that after treatment, there is a significant decrease in the thickness of the film due to NaOH etching (Figure 1a,d), which was further corroborated by the appearance of creases on the initially smooth surface of PET (Figure 1b,e). Furthermore, the decrease in the water contact angle (Figure 1c,f) indicates an increase in the surface hydrophilicity, which was attributed to the additional –COOH moieties generated after surface modification.

Analysis of the amount of –COOH on the film was performed using a solution of TBO, which is known to form an equimolar complex with the carboxylic acid.^38^ The complexed –COOH groups were then desorbed by exposing the films to acetic acid, which was subsequently analyzed by UV–vis to indirectly quantify the amount of carboxylic acid groups per surface area of the film.

As shown in Figure S1a, the TBO solution has an absorbance maximum at 633 nm. UV-Vis analysis at this wavelength for different TBO standard solutions allowed for the construction of a calibration curve (Figure S1b) to quantify the amount of –COOH. From Table S1, it can be gleaned that KMnO_4_ treatments generally produced more –COOH than UV/H_2_O_2_ treatments, with the 80 min treatment with KMnO_4_ generating the greatest number of –COOH groups. This is also visually indicated by the more intense blue coloration of the film (Figure S1c) upon treatment with TBO, proving that more carboxylic acid groups were available for complexation. Prolonged oxidation period, however, leads to possible decarboxylation,^36^ hence resulting in a decrease in the number of –COOH. Also, the low turnout for H_2_O_2_/UV treatment can be attributed to the low radiation intensity of the UV source, which leads to a lower number of generated radicals that would cause PET oxidation.^37^ Based on these results, oxidation with KMnO_4_ was then utilized for the succeeding PET treatments in this study.

### 3.2 Fabrication and Characterization of the Porphyrin-Grafted PET Films

With the PET films already enriched with the –COOH groups, a water-soluble oxazoline-functionalized polymer was spin-coated onto the film and subsequently heated to facilitate the attachment of the polymer, as illustrated in Scheme S1. During the process, oxazoline undergoes a ring-opening addition reaction with the carboxylic acid groups on the surface, as shown in Scheme S2.^39^ This reaction results in a strong covalent attachment consisting of an amide-ester linkage, leading to a more stable immobilization. The remaining oxazoline moieties on the spin-coated polymer subsequently served as anchoring points for the attachment of the carboxylic acid groups of TCPP, utilizing the same oxazoline-carboxylic acid reaction, which finally afforded the pale brownish pink film (Scheme S1). The significance of the oxazoline polymer as an intermediate material for TCPP grafting is further explored in Figure S2, while additional optimizations concerning the time of grafting of TCPP are shown in Figure S3.

**Figure 2:**
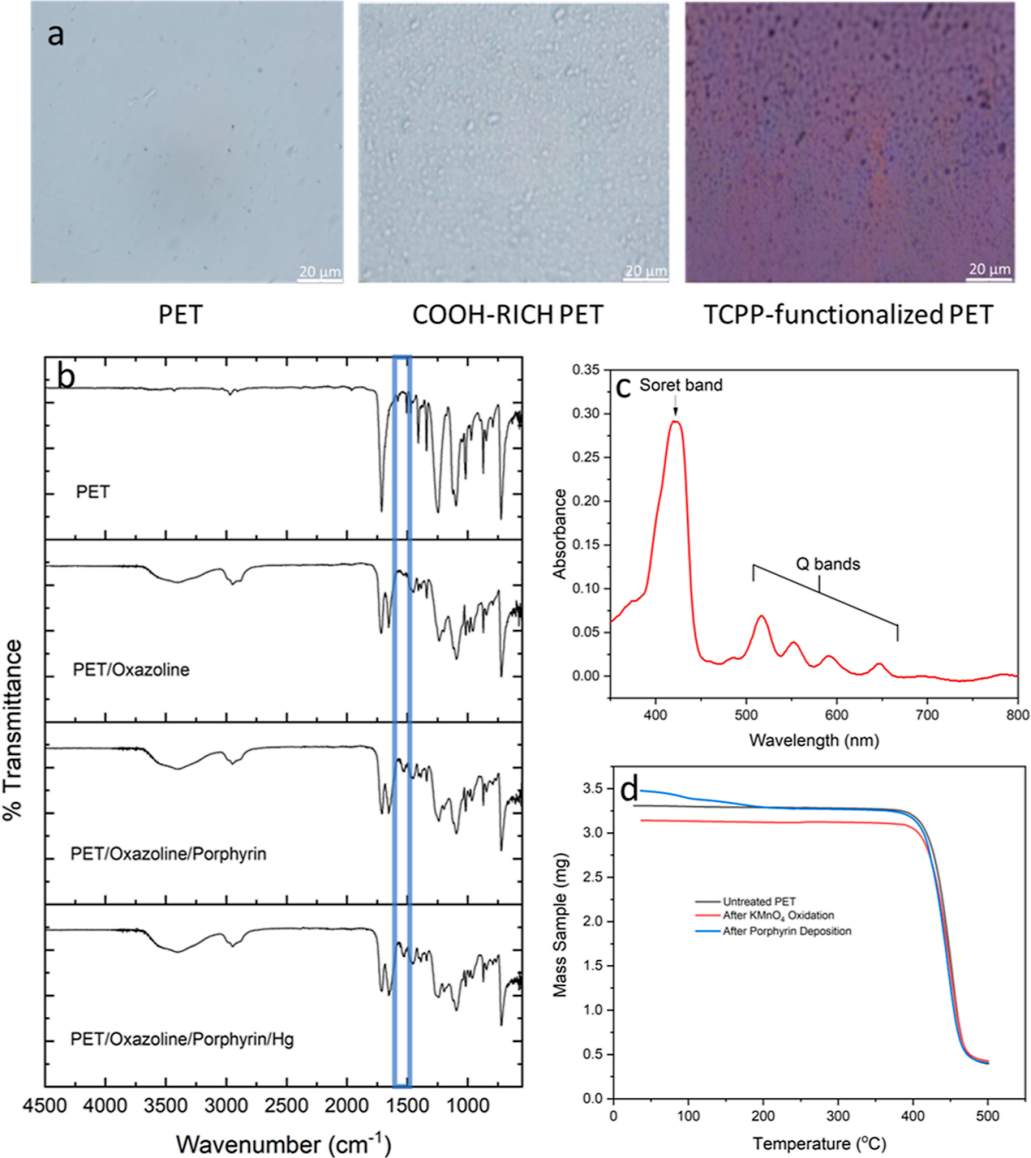
(a) Microscopy images of PET films after every treatment, (b) IR profiles of PET after oxazoline attachment and TCPP grafting, (c) UV–vis profile of the final probe, and (d) thermal stability data.

The changes in the morphologies of the PET films after every step of the surface functionalization are shown visually in the microscopic images in Figure 2a. As observed, the surface of the film became rougher due to the etching process. Additionally, the succeeding treatment with porphyrin rendered the film fully colored suggesting uniform grafting of the material. The IR data in Figure 2b further corroborates the successful grafting of porphyrin, as indicated by the presence of a small peak at 1530 cm^–1^. Also, the sharp peaks at 1720 and 1650 cm^–1^ are attributed to the amide and ester linkages upon reaction of the oxazoline moieties with the carboxylic acids.^40^ The porphyrin grafting onto the surface is further verified by the UV–vis profile of the final product, as revealed in Figure 2c, in which the peaks intrinsic to the free-base porphyrin are observed. These are the strong Soret band at 422 nm and four weak Q bands between 500 and 700 nm.^26,41^ Moreover, the thermogravimetric analysis data in Figure 2d show that despite the series of treatments in fabricating the probe, the final product’s degradation profile is similar to that of the initial PET film, hence confirming that the material is thermally stable. Lastly, the thickness measurements through SEM of the spin-coated oxazoline polymer and the resulting film are presented in Figure S4.

### 3.3 Probe Operation and Regeneration

**Scheme 2:**
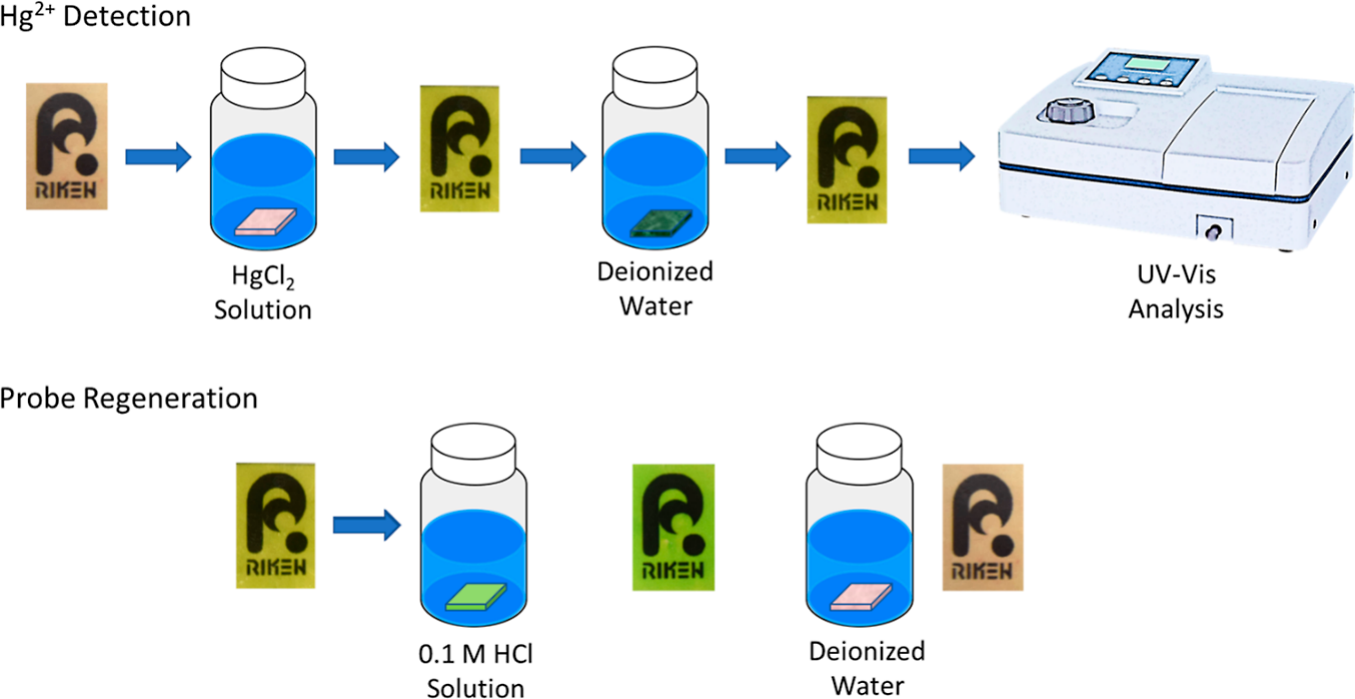
Probe Operation and Regeneration (Adapted with Permission to Use the Logo Copyrighted by RIKEN)

The colorimetric probe operation is very straightforward and involves only submerging the film in an aqueous solution of 0.01 M HgCl_2_ and washing it subsequently with deionized water. After a brief agitation period, it was observed that the film gradually changed color from pale brownish pink to green, as illustrated in Scheme 2, which indicates the chelation of the Hg^2+^ ions by the grafted porphyrin moieties on the film. Due to the probe’s transparency, UV–vis analysis of the films can then be performed to quantify the amount of complexed Hg^2+^ ions. Thereafter, the probe could be easily regenerated by shaking it in a dilute HCl solution for 30 s and further washing it with deionized water. During this process, the acid protonates the pyrrole groups in the porphyrin ring, which causes the expulsion of the metal ion from the core.^42^ The reversion of the film’s color to its initial pale brownish pink state (green to pink) indicates that it could already be used for another round of Hg^2+^ detection.

### 3.4 Characterization of the Metal-Bound Colorimetric Probe and Sensing Mechanism

**Figure 3:**
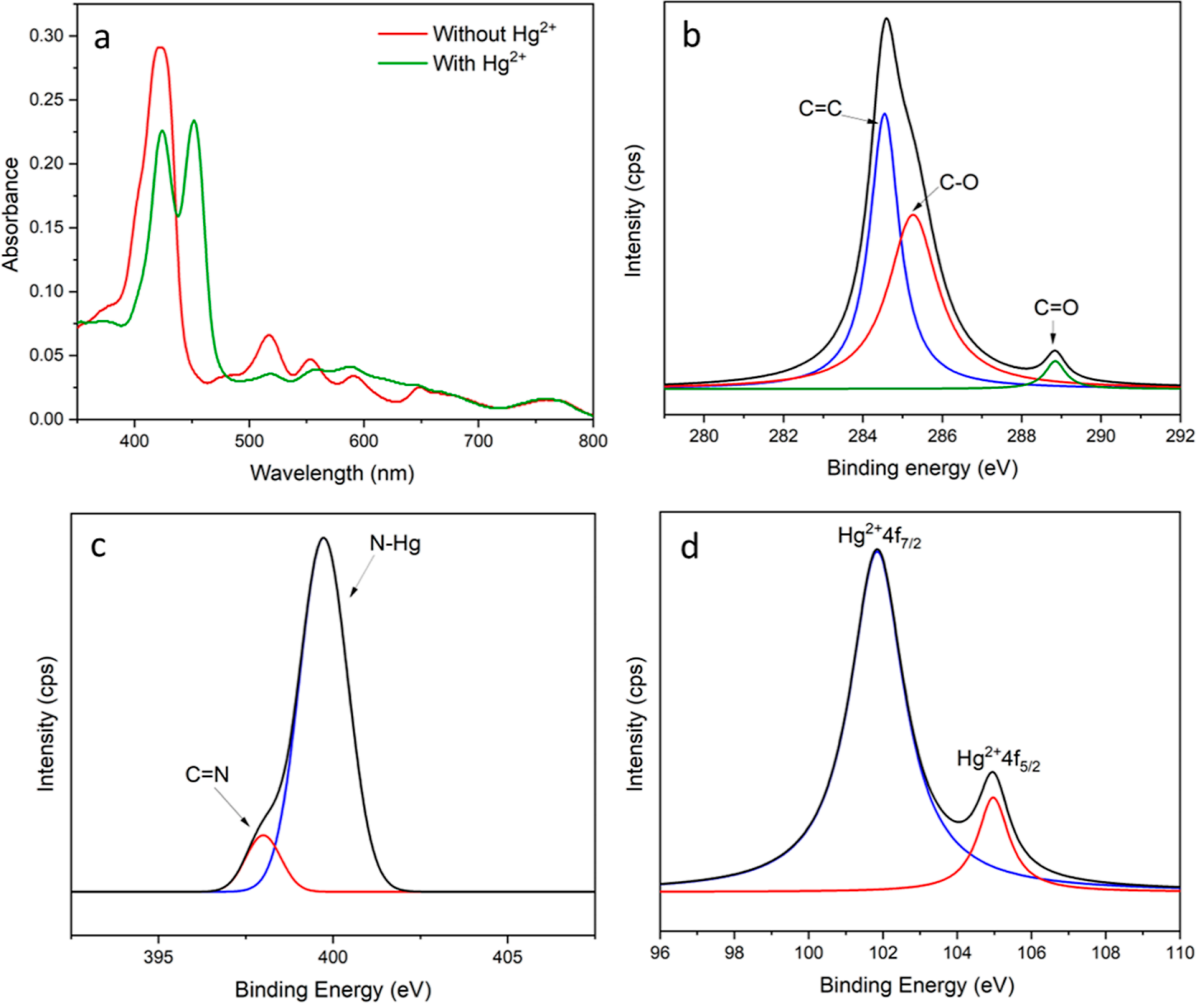
Characterization of the Hg^2+^-bound colorimetric probes. (a) UV–vis spectra of the free and metal-bound colorimetric probe; deconvoluted XPS spectra of the (b) C 1s, (c) N 1s, and (d) Hg 4f core levels for the metal-bound colorimetric probe.

Upon exposure of the probe to a dilute solution of HgCl_2_, accompanying changes in its coloration and spectral properties were observed. As shown in Figure 3a, the complexation of Hg^2+^ by porphyrin resulted in a decrease in the Soret band intensity with a concomitant appearance of a new red-shifted peak at 451 nm. The four Q bands also appeared broader and were no longer as resolved as before Hg^2+^ complexation.^33,43^ Monitoring the new peak at 451 nm allowed for quantification of the concentration of Hg^2+^ in the aqueous solution upon UV–vis calibration.

The spectral change may be attributed to the formation of an out-of-plane complex between Hg^2+^ and porphyrin. The large size of the Hg^2+^ ion prevented it from being inserted into the macrocyclic core; hence, the coordination bond formed between the two resulted in an out-of-plane configuration. This conformation causes a distortion in the planar structure of the ring, which significantly contributes to the change in its spectral properties.^42^ In addition, out-of-plane complexes are kinetically labile, which renders both the association and dissociation of metal ions faster as compared to in-plane complexes,^44,45^ making them suitable for reusable sensing applications. This out-of-plane configuration may result to 1:1 and 3:2 Hg^2+^-to-porphyrin ratios, which are greatly dependent on the concentrations of both porphyrin and Hg^2+^.^44,46^ The greater amount of free porphyrins also results in the aggregation of the macrocyclic rings, depicted by the broad and unresolved peaks in the UV–vis spectrum of the free base, particularly in the Q-band region.^34^ To investigate the possible aggregation of porphyrin moieties upon immobilization on the PET surface, the UV–vis spectrum of the probe was compared with that of a 10^–6^ M TCPP solution in 0.01 M NaOH, as shown in Figure S5. Upon an increase in the concentration of TCPP, there was a significant broadening of both the Soret and Q bands, indicating the aggregation of porphyrin moieties (Figure S5a). Furthermore, at a concentration of 10^–4^ M, a new red-shifted peak was observed beside the Soret band, consistent with the findings by Liu et al.^47^ In their paper, they noted that at concentrations less than 6.0 μM, the TCPP exists as a monomeric species, which is similar to our current findings. Comparing the UV–vis profile of the fabricated probe with that of 1 μM TCPP (Figure S5b), it was observed that the Soret band of the film is slightly red-shifted to 422 nm as compared to that of TCPP, which is common among immobilized porphyrin moieties.^34^ Furthermore, both profiles have sharp Soret peaks, implying no aggregation among the porphyrin groups upon immobilization on the PET substrate. Interestingly, the Q bands for the probe are even more resolved than those of the TCPP solution, which further verifies the presence of porphyrin as monomeric species on the film. This may be attributed to the covalent attachment of individual porphyrin moieties to the oxazoline units on the PET surface, which served as spacers that hinder the macrocycles from diffusing freely and aggregating, hence easier interaction with the Hg^2+^ ions. Moreover, the ratio of Hg^2+^-to-porphyrin upon complexation was also investigated through UV–vis analysis. Here, the UV–vis profiles of low and high-concentration TCPP solutions upon exposure to increasing amounts of Hg^2+^ were compared with that of the probe in Figure S6. For both the high- and low-concentration porphyrin solutions, increasing the concentration of Hg^2+^ caused a gradual decrease in the Soret band at 414 nm with the concomitant appearance of a red-shifted peak at 456 nm. This bathochromic shift is indicative of the out-of-plane complex formed between Hg^2+^ ion and porphyrin while the large magnitude of the shift suggests the presence of a 3:2 Hg^2+^-to-porphyrin ratio. It is interesting to note that unlike previous literature, the presence of a 1:1 complex, which is characterized by a slight shift in the Soret band,^34^ was not observed. This can be explained by the observations in some papers where 1:1 complexes were only detected at porphyrin concentrations lower than 10^–6^ M.^34,44^ Additionally, the absorbance profile of the probe in Figure S6c closely resembled that of the low-concentration porphyrin, which is ascribed to the absence of porphyrin aggregations that cause the broadening of the peak that is present in high-concentration porphyrin solutions. This resemblance indicates that the Hg^2+^ ions and porphyrin moieties on the film form a 3:2 complex, which is highly similar to the findings reported by the groups of Valicsek^44^ and Adeyemo.^46^ With these inferences, quantification of the number of TCPP grafted on PET was conducted. The films were submerged in a solution of HgCl_2_ for an extended period to ensure that all porphyrin rings are metalated with Hg^2+^ ions. The complexed heavy metal ions were then detached from the film via treatment with 2% HNO_3,_ which were subsequently analyzed by ICP–MS. Since Hg^2+^ ions undergo complexation with porphyrin in a 3:2 ratio, the amount of grafted TCPP was then calculated to be 51.3 nmol/cm^2^.

XPS studies were also conducted to provide additional insights into the complex formed between mercury and porphyrin grafted on PET. As shown in Figure 3b, the C 1s spectrum of the Hg-bound film was deconvoluted into three constituent peaks. The peak at 284.6 eV is attributed to the C=C carbons of the porphyrin rings, while the peaks at 285.3 and 288.8 eV can be ascribed to the C–O and C=O functional groups present in the material, respectively.^49^Figure 3c shows the deconvoluted N 1s spectrum where two peaks were clearly observed. The large peak at 399.70 eV may be assigned to the N–Hg bond formed upon complexation, indicating that both the aza and deprotonated pyrrolic nitrogens at the macrocyclic center have interacted with the Hg^2+^.^50^ The small peak at 398 eV, on the other hand, represents the aza (C=N) nitrogen for the unreacted oxazoline moieties.^51^ Furthermore, the presence of Hg^2+^ ions was verified and is shown in Figure 3d, which revealed two peaks in the Hg 4f XPS spectrum. These peaks at 101.5 and 105.0 eV are attributed to Hg^2+^ 4f_7/2_ and Hg^2+^ 4f_5/2_, respectively, resulting from the spin orbit splitting of the 4f orbital. These data further proved the absence of Hg(0) in the complex as it would have elicited a peak at 100 eV.^52,53^ Lastly, XPS images of atomic percent distribution of the C 1s, N 1s, and Hg^2+^ 4f are presented in Figure S7, which further verify that the porphyrin is homogeneously distributed on the surface of the film and that Hg^2+^ ions are attached to the rings.

### 3.5 Selectivity Studies of the Porphyrin-Grafted PET Films

**Figure 4:**
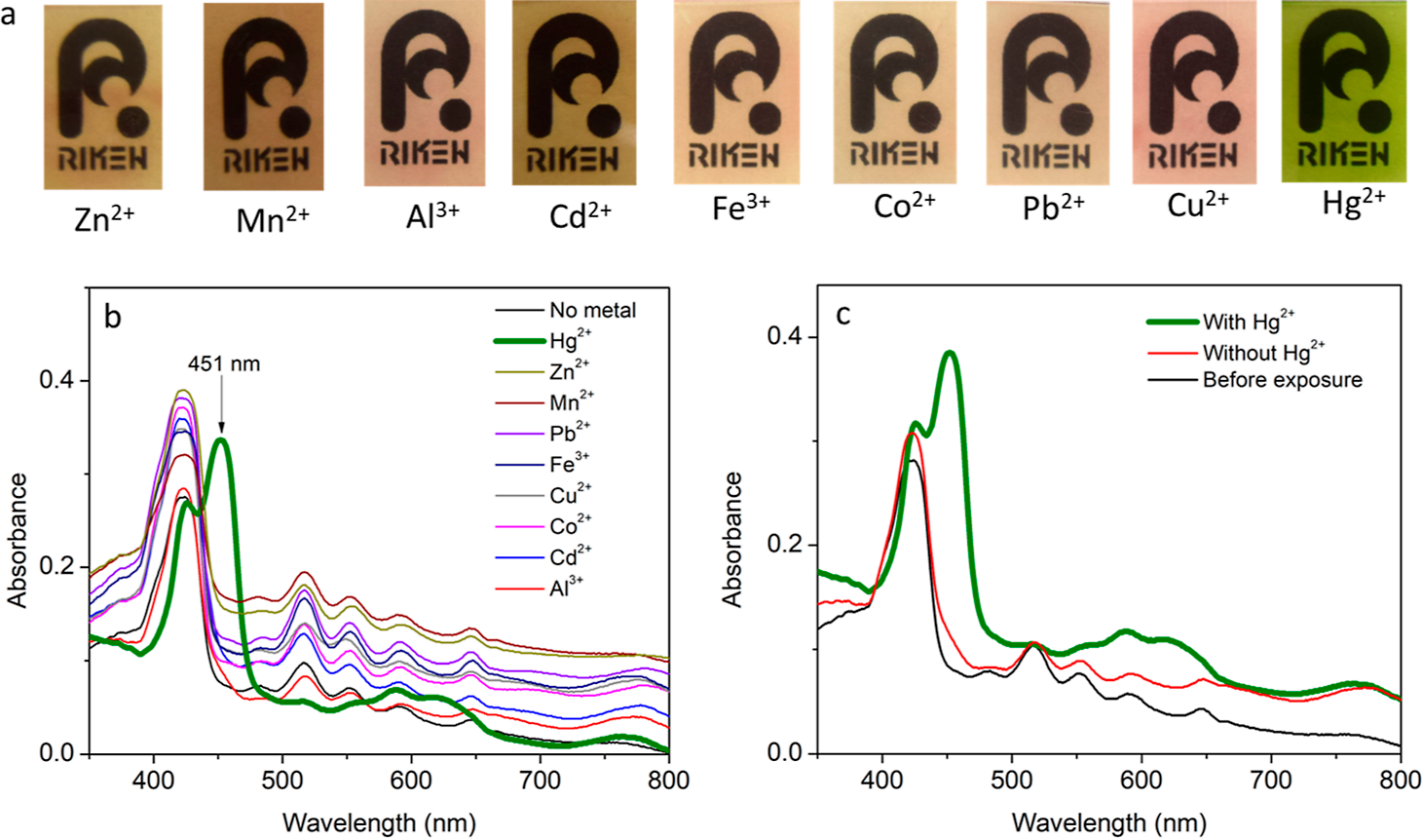
(a) Color of the films after exposure to different metal ions, (b) UV–vis profiles of the film after exposure to each metal ion, and (c) UV–vis profiles of composite mixtures of metal ions with and without Hg^2+^ (adapted with permission to use the logo copyrighted by RIKEN).

The selectivity of the probe to Hg^2+^ was also evaluated against heavy metal ions such as Pb^2+^, Cd^2+^, Zn^2+^, Mn^2+^, Al^3+^, Fe^3+^, Co^2+^, and Cu^2+^. This was conducted by immersing the material in a 0.01 M buffered solution (pH 6.8) of each ion and subsequently agitating for 2 h. By visual inspection, it was observed that none of the ions manifested any change in the color of the film even after prolonged exposure, except in the case of Hg^2+^ where the film underwent color change within less than 5 min, as shown in Figure 4a. This was corroborated by the UV–vis analysis of the films (Figure 4b), in which all the ions except for Hg^2+^ had the same profile as the nonmetalated probe. It was solely the Hg^2+^ ion that exhibited a different spectrum with a distinct strong peak at 451 nm, which is indicative of Hg^2+^ attachment to the metal center. To further verify the selectivity of the colorimetric probe, two composite solutions consisting of 0.01 M of each of the initially tested ions were prepared; one of the mixtures was spiked with 0.001 M Hg^2+^ while the other was not. From this competition experiment, it was observed that only the composite solution spiked with Hg^2+^ resulted in a visible change in the color of the film to green, thereby further demonstrating the high selectivity of the film toward mercuric ions even in the presence of several interferences. This was further supported by the UV–vis profiles, as shown in Figure 4c where a mixture of several metal ions spiked with Hg^2+^ elicited a strong peak at 451 nm. This investigation is more comprehensive than other previously studied systems as we have tested a large number of possible interferences that could potentially affect the material’s performance, hence, indisputably confirming our probe’s selectivity toward Hg^2+^ detection.

### 3.6 Effect of pH on the Colorimetric Probe

**Figure 5:**
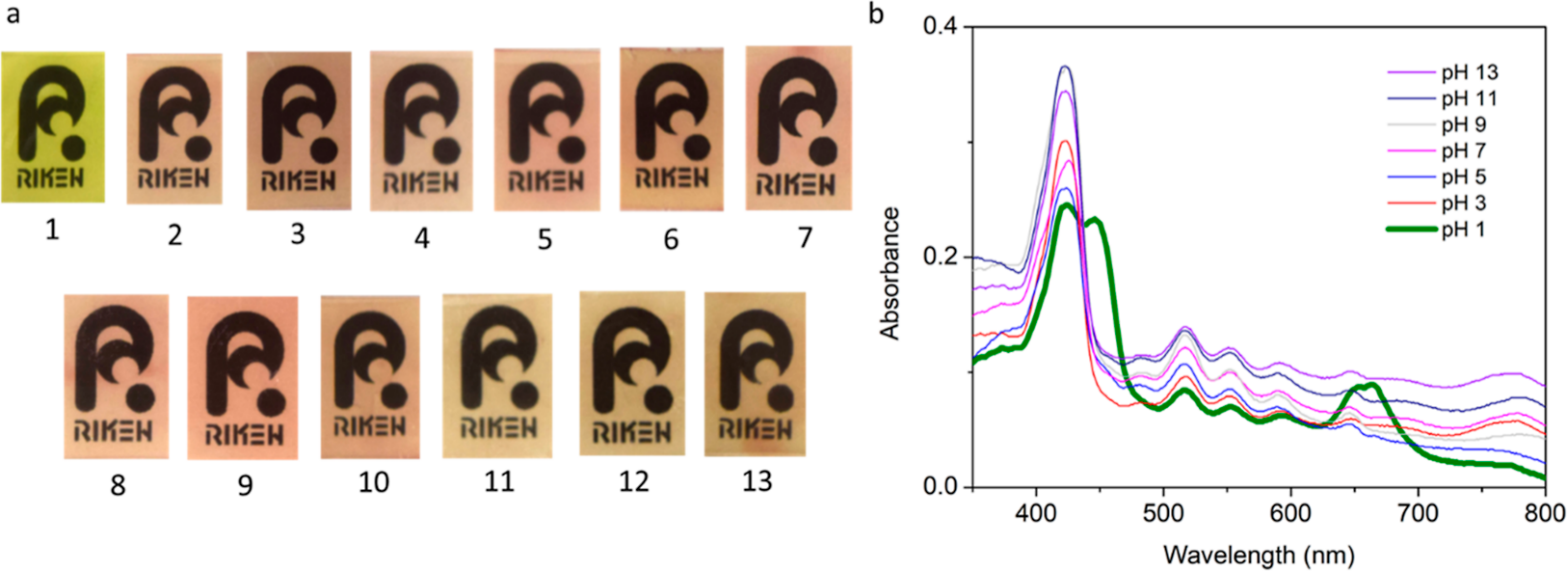
(a) Color of the films after exposure to different pH conditions. (b) UV–vis profiles of the film after exposure to aqueous solutions with different pH values (adapted with permission to use the logo copyrighted by RIKEN).

Since it is known that porphyrin may undergo protonation or deprotonation upon exposure to different pH conditions,^32,54,55^ it is necessary to investigate the effect of varying pH on the porphyrin-grafted probes and their absorbance profiles. In our experiments, the films were immersed and then shaken for 2 h in solutions adjusted to specific pH values ranging from 1 to 13. By visual inspection (Figure 5a), it can be inferred that only the film exposed to pH 1 solution transitioned to green while the rest were generally unaffected by the other pH conditions. The change in the color of the film is indicative of the protonation of the nitrogen present in the porphyrin center, which results to the repulsion of the four protons in the ring, thus causing a strain in its planarity.^42^ This is supported by the UV–vis profiles of the films, in which the one exposed to pH 1 manifested a different profile as opposed to the rest, which exhibited the same UV–vis characteristics as those of the untreated film, as shown in Figure 5b. Furthermore, the films retained their sensing ability when exposed to Hg^2+^ ions after the pH treatment. Based on these observations, we have ascertained that the film can be exposed to a very broad pH range without compromising its ability to detect Hg^2+^ ions. The results, therefore, indicate the suitability of the material for mercury ion detection in various water sources, which typically have pH values between 5.5 and 8.^56^ It is worth noting, however, that very acidic samples can give false positive results for mercury contamination due to the green coloration of the films. This can be easily addressed by washing the film with deionized water until the pH becomes neutral, as shown in Scheme 2. Reversion of the film to the initial faint brownish pink state indicates that the green coloration is due to the high acidity of the sample, while the persistence of the green coloration even after washing confirms the presence of Hg^2+^. The capability of the film to withstand varying pH conditions is further highlighted in Table S2, where our material registered the widest operating pH range among all of the sensors listed. This underscores our material’s practicability in testing bodies of water with different pH conditions.

### 3.7 Response Time, Sensitivity, and Quantification

The duration required for a visible change in the color of the film was also studied. Based on Table 1, the more concentrated the aqueous solution of Hg^2+^ is, the faster it transitions to green. Also, the longer exposure of the film results in a deeper green color. However, for extremely low concentrations, like 10^–8^ M, no visible change in the color of the films was observed even after submerging the films for more than 2 days. This may be attributed to the lack of sufficient Hg^2+^ ions present in the solution to complex with the available porphyrin moieties on the surface that could elicit a visible color change. This may be offset by utilizing a large quantity of the solution having the same concentration, thereby providing more ions that can be trapped within the probe. Based on our results, the material can manifest a visible color transition until 10^–7^ M after a day of exposure, thereby demonstrating our materials’ high sensitivity for the qualitative assessment of mercuric ion contamination in aqueous samples. Our findings are also comparable with those of Zhang and co-workers,^32^ in which they developed nanofibers containing porphyrin that can respond to Hg^2+^ with a limit of detection at 10^–7^ M (20 pbb) using the naked eye.

**Table 1:** Response Times of the Porphyrin-Grafted PET Colorimetric Probe

concentration (M) in 100 mL solution	response time
10^–2^	<1 min
10^–3^	3 min
10^–4^	12 min
10^–5^	30 min
10^–6^	6 h
10^–7^	24 h

The actual amount of mercury in aqueous samples may also be quantified through UV–vis analysis of the films. In our experiments, standard solutions with varying concentrations of the mercuric ion were prepared and then shaken for 10 min with the probes immersed in them. Subsequently, a calibration curve was plotted with respect to the films’ absorbance at 451 nm. From Figure S8a, it can be observed that the absorbance increases with concentration but gradually plateaus at higher amounts of Hg^2+^. This may be explained by the number of vacant porphyrin moieties that are available to interact with the ion. At higher Hg^2+^ concentrations, most of the porphyrins have already been metalated, so a further increase in the ion concentration no longer translates tothe same degree of green coloration and absorbance.

Cognizant of this, absorbances at lower concentrations were utilized in constructing the calibration curve, as shown in Figure S8b, where a linear correlation between the two parameters was obtained. While the UV analysis of the films can detect signals for 10^–8^ M, which is the LOD of the probe, the linear range of the calibration curve was ascertained to be from 3.7 × 10^–6^ M (0.739 ppm) to 1.4 × 10^–3^ M (295.5 ppm). Spiked samples containing 59.10 and 221.63 ppm of Hg^2+^ were also prepared to test the reliability of the method, and it was found out that the probe was able to detect 50.33 and 208.33 ppm, which translated to a % recovery of 85.16 and 94.00%, respectively. Based on the LOD, our material can detect concentrations that are within the maximum residue limit allowed by the WHO (3 × 10^–8^ M),^34^ highlighting its outstanding ability for Hg^2+^ monitoring in potable and other water sources. The LOD of our fabricated probe is also comparable to and even better than those of other sensing materials previously developed (Table S2) in the detection of Hg^2+^, while enjoying the advantages of facile fabrication and easy operation.

### 3.8 Reusability and Applicability to Real Samples

**Figure 6:**
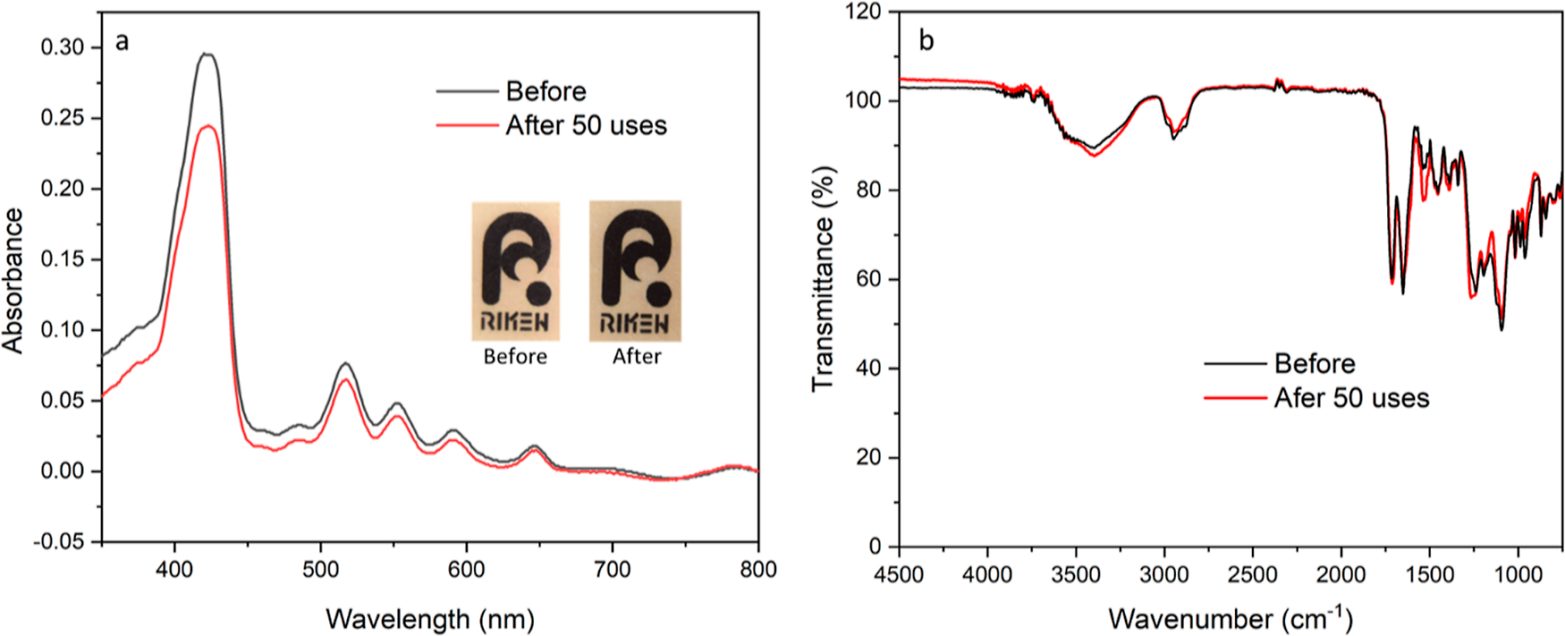
Characterization of the film before and after reusability experiments. (a) Actual photographs and UV–vis profiles and (b) FT-IR spectra of the film (adapted with permission to use the logo, copyrighted by RIKEN).

Another important parameter that characterizes the practicability of a colorimetric probe is its reusability. The fabricated porphyrin-grafted PET film can easily be regenerated by treating the metalated (with bound mercury) probe with dilute HCl and subsequently washing it with copious amounts of water to revert the film to its original faint brownish pink color (unbound state), as depicted in Scheme 2. After regeneration, it can then be used immediately for the next sensing experiment. As shown in Figure 6a, the film’s original color has not changed significantly, suggesting that it has retained its initial properties despite its continuous usage. This is further verified by the similarity in the film’s UV–vis and FT-IR profiles before and after 50 cycles, as shown in Figure 6a and b.

**Figure 7:**
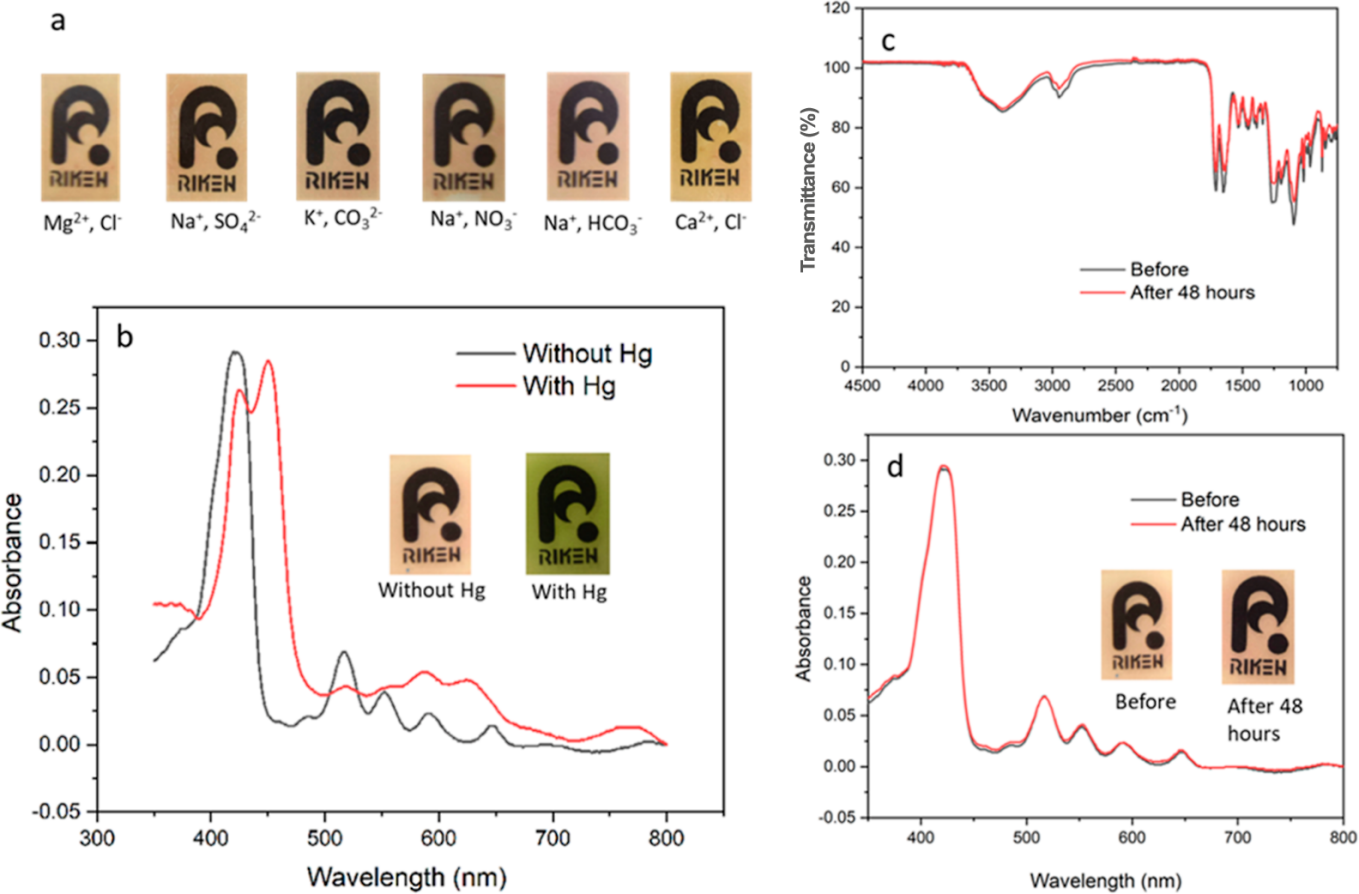
(a) Images of the films when exposed to common ions present in river samples, (b) UV–vis spectra of films exposed to Hg^2+^-spiked and nonspiked river samples, and (c) FT-IR and (d) UV–vis profiles of the probe before and after exposure to river samples for 48 h (adapted with permission to use the logo copyrighted by RIKEN).

The suitability of the film for real sample analysis was also investigated by first exposing the material to common interferences present in actual river samples. The films were immersed in 10^–2^ M solutions of Mg^2+^, Na^+^, K^+^, Ca^2+^, NO_3_^–^, HCO_3_^–^, Cl^–^, CO_3_^2–^, and SO_4_^2–^ and then shaken for 2 h. We wanted to determine whether these ions can interact with the film, potentially affecting the quality of the sensing process. From Figure 7a, the images revealed that the films remained unchanged even after prolonged exposure to these interferences. We also tested the films using actual river samples collected from the Arakawa river in Wako City, Japan. The films were exposed and shaken for an extended period in Hg^2+^-spiked and nonspiked river water samples. The generated UV–vis profiles and the images of the films (Figure 7b) are consistent with the data from the spiked and nonspiked standard Hg^2+^ solutions in the previous section, hence verifying the applicability of the films for real-sample analysis. Moreover, the film’s stability was investigated for any signs of degradation when exposed to actual river samples for 48 h. Based on the UV–vis and IR profiles in Figure 7c,d, it was confirmed that the films retained their initial features with no signs of degradation after the stability test. This was also verified by the similarity in the film’s appearance before and after 48 h of exposure. These findings for the reusability, stability, and applicability to actual river samples further emphasize the probe’s superior performance as compared to other materials listed in Table S2. Only one other probe was able to withstand the continuous reusability experiments for 50 cycles among all the materials listed. Furthermore, testing with a high concentration of possible ions in water ensured that the probe could operate reliably, even under different sample conditions.

## 4 Conclusions

In summary, we developed a selective, stable, and reusable porphyrin-grafted PET film for the colorimetric detection of the Hg^2+^ ions in aqueous samples. This material takes advantage of both the metal-sensing ability of porphyrin and the robustness of PET as stable, flexible, and transparent substrate in the development of the colorimetric probe. The porphyrin-grafted PET was fabricated through a facile approach, in which an oxazoline-based polymer both served as a secure adhesive to the PET and as anchoring points for porphyrin grafting. Besides immobilizing the porphyrin units on the surface of the substrate, the oxazoline polymer also functioned as a spacer, preventing aggregation of the porphyrin moieties. Sensing experiments revealed that the film, by virtue of the porphyrin groups, visibly transitions from a faint brownish pink to green upon complexation with the Hg^2+^ ions in aqueous samples. This property, together with the PET substrate’s flexibility and stability, renders the material suitable for the onsite and straightforward “naked-eye” detection of mercury ions at concentrations as low as 10^–7^ M (20 ppb). Moreover, the film’s transparency allowed the quantification of mercury ions by UV–visible analysis, which can detect as low as 10^–8^ M of Hg^2+^ ions and is within the maximum residue limit set by the WHO for drinking water. The material was also proven to be highly selective for mercuric ions, even in the presence of numerous interferences and other metal ion analogues at high concentrations. Additionally, the film can operate over a very wide pH range and can be easily regenerated for repeated usage at least 50 times, without any observable decline in its sensing ability. All these remarkable properties exemplify our material’s excellent potential as a mercury (II) ion colorimetric probe in the onsite qualitative and lab-based quantitative analysis of various water samples.

## Supporting Information Available

Materials, carboxylic acid quantification, film fabrication, optimization and additional characterizations, and mercuric ion quantitative analysis (PDF)
