# Supplementary material for: Porphyrin-Grafted Poly(ethylene terephthalate) as a Reusable and Highly Selective Colorimetric Probe for Mercuric Ion Contaminants in Aqueous Samples
Source: ACS Appl Mater Interfaces. 2024 Jul 22;16(30):39195–205. doi: 10.1021/acsami.4c03846 (PMC11299150; doi:10.1021/acsami.4c03846)
Supplement: Supplementary file 1 — am4c03846_si_001.pdf [file am4c03846_si_001.pdf]

Supporting Information

Porphyrin-Grafted Poly(ethylene terephthalate) as a Reusable and Highly Selective Colorimetric Probe for Mercuric Ion Contaminants in Aqueous Samples

Eduardo C. Atayde Jr.[1,2,3,4], *Yasumasa Takenaka* [4*], *Hideki Abe* [4], *Ming-Rou Wu* [3] *and Kevin C.-W. Wu* [1,3,5,6*]

[1] Molecular Science and Technology Program, Taiwan International Graduate Program, Academia Sinica, No. 128, Sec. 2, Academia Road, Taipei 11529, Taiwan

[2] Department of Chemistry, National Tsing Hua University No. 101 Sec. 2, Kuang-Fu Road, Hsinchu 30013, Taiwan

[3] Department of Chemical Engineering, National Taiwan University, No. 1, Sec. 4, Roosevelt Road, Taipei 10617, Taiwan

[4] Bioplastic Research Team, RIKEN Center for Sustainable Resource Science
2-1 Hirosawa, Wako, Saitama 351-0198, Japan

[5] Department of Chemical Engineering and Materials Science, Yuan Ze University, No. 135, Yuandong Rd., Zhongli District, Taoyuan 32003, Taiwan

[6] Department of Chemical Engineering, Chung Yuan Christian University, No. 200, Zhongbei Rd., Zhongli Dist., Taoyuan 320, Taiwan

*Correspondence: kevinwu@ntu.edu.tw; yasumasa.takenaka@riken.jp

Contents **Page**

Contents	Page
Chemicals and Reagents	S3
Figure S1. COOH Quantification	S4
Table S1. Amount of the generated carboxylic acid groups per treatment	S4
Scheme S1. Fabrication of porphyrin-grafted PET	S5
Scheme S2. Reaction of carboxylic acid with oxazoline	S5
Figure S2. Grafting of untreated and treated PET films with oxazoline	S6
Figure S3. TCPP grafting on oxazoline-coated PET at different times	S7
Figure S4. Thickness measurements by SEM	S8
Figure S5. UV-Vis spectra of TCPP solutions and the fabricated probe	S8
Figure S6. UV-Vis spectra of the TCPP solutions and fabricated probe upon treatment with increasing Hg^{2+} concentration	S9
Figure S7. XPS images of the atomic percent distribution	S10
Figure S8. Hg^{2+} quantification using the probe	S10
Table S2. Comparison of the fabricated probe's performance with other Hg^{2+} sensors	S11-S12
References	S13-S16

Chemicals and Reagents

Tetrakis(4-carboxyphenyl)porphyrin (TCPP, TCI, >97%), oxazoline functional polymer (EPOCROS™ WS-700, Nippon Shokubai), toluidine blue (TBO, TCI, >99.0%), iron (III) chloride ($FeCl_3$, Fujifilm, 99.0%), manganese chloride ($MnCl_2$, Fujifilm, >99.0%), mercury (II) chloride ($HgCl_2$, Kanto Chemical, >99.5%), copper (II) chloride dihydrate ($CuCl_2 \cdot 2H_2O$, Kanto Chemical, >99.0%), lead (II) nitrate ($Pb(NO_3)_2$, Kanto Chemical, >99.5%), cobalt (II) chloride anhydrous ($CoCl_2$, Kanto Chemical, >95%), cadmium (II) chloride 2.5-hydrate ($CdCl_2 \cdot 2.5H_2O$, Kanto Chemical, >95%), super dehydrated N,N- dimethylformamide (Kanto Chemical, >99.5%), sodium hydroxide (NaOH, Nacalai Tesque, >97.0%), sodium sulfate (Na_2SO_4, Fujifilm, >99.5%), magnesium chloride, ($MgCl_2$, Fujifilm, >97%), potassium carbonate, (K_2CO_3, Fujifilm, >99.5%), sodium nitrate, ($NaNO_3$, Fujifilm, >98%), sodium bicarbonate ($NaHCO_3$, Fujifilm, >99.0%), calcium chloride ($CaCl_2$, Kanto Chemical, >95%), potassium permanganate (TCI, >99.5%), hydrochloric acid (HCl, Nacalai Tesque, 35%), sulfuric acid (H_2SO_4, Nacalai Tesque, 97%), nitric acid (HNO_3, Nacalai Tesque, 60%) and acetic acid (CH_3COOH, Nacalai Tesque, >99%) were used as received without further purification. PET films (Lumirror® T60 Transparent, 100μm thickness) were cut to 2 cm x 2 cm and were washed thoroughly with deionized water and acetone before use. All solutions were prepared using deionized water (Millipore Milli-Q system) throughout this study.

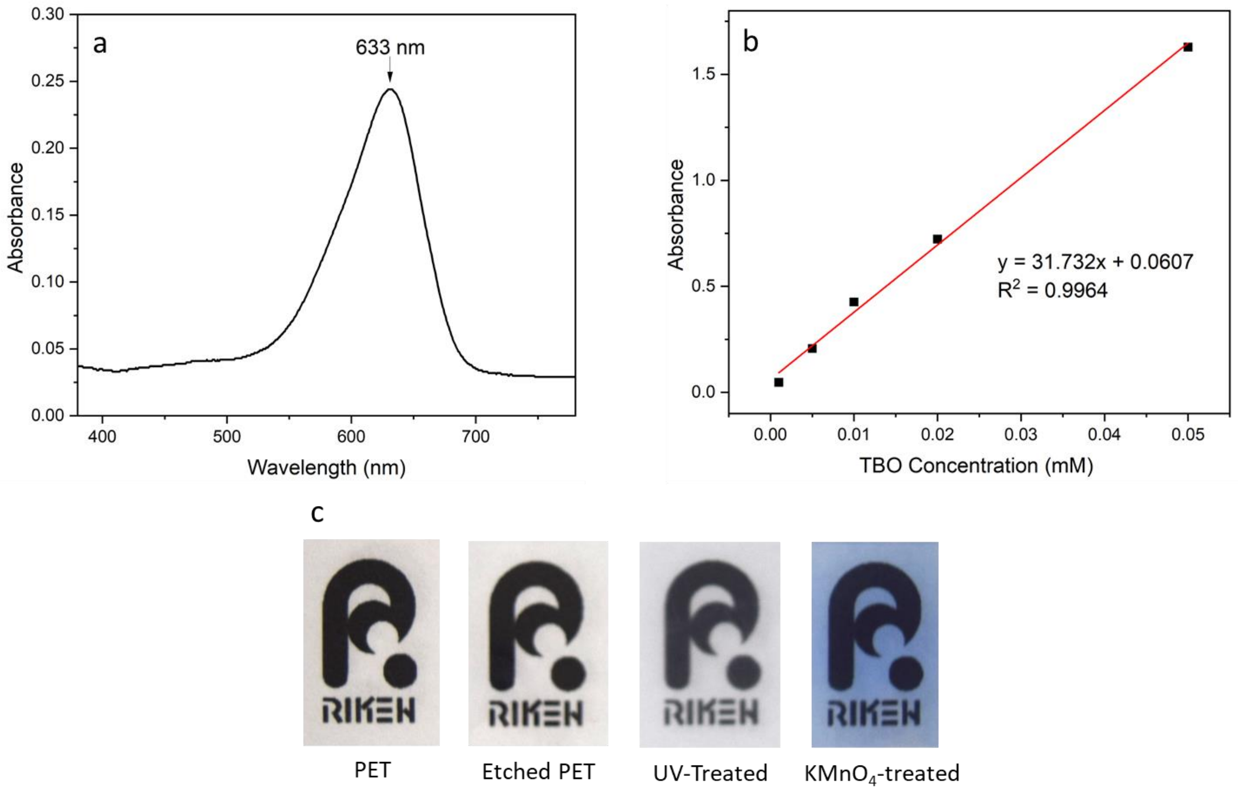

Figure S1. (a) The absorbance profile of TBO, (b) calibration curve for -COOH quantification and (c) actual film colors after exposure to TBO. (Adapted with permission to use the logo copyrighted by RIKEN.)

***Table S1**. Amount of the generated carboxylic acid groups per treatment*

PET Samples	Amount of TBO (mM)	Amount of COOH Groups (nmol/cm^2)
PET	0	0
Etched PET	0.0189	0.0013
UV/H_2O_2, 1.5 h	0.4601	0.0322
UV/H_2O_2, 3 h	0.5862	0.0410
$KMnO_4$, 60 ^{o}C, 20 min	11.721	0.8222
$KMnO_4$, 60 ^{o}C, 80 min	62.0383	4.3444
$KMnO_4$, 60 ^{o}C, 120 min	56.8700	3.9825

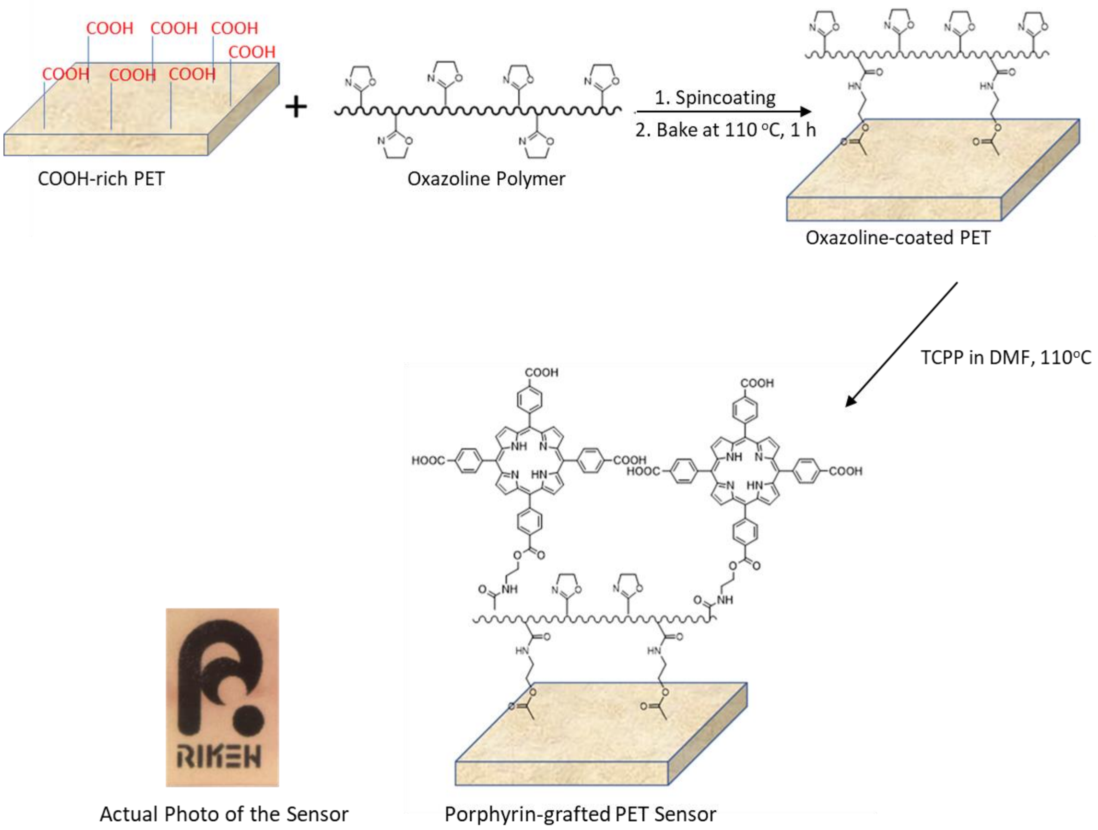

Scheme S1. Fabrication of porphyrin-grafted PET films using oxazoline polymer as covalent adhesive (Adapted with permission to use the logo copyrighted by RIKEN).

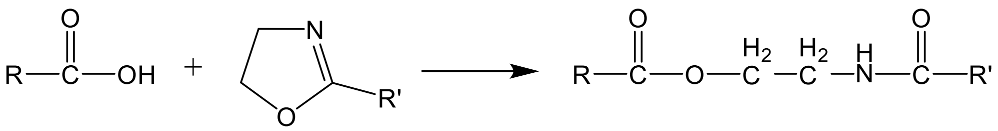

Scheme S2. Reaction of carboxylic acid with oxazoline.

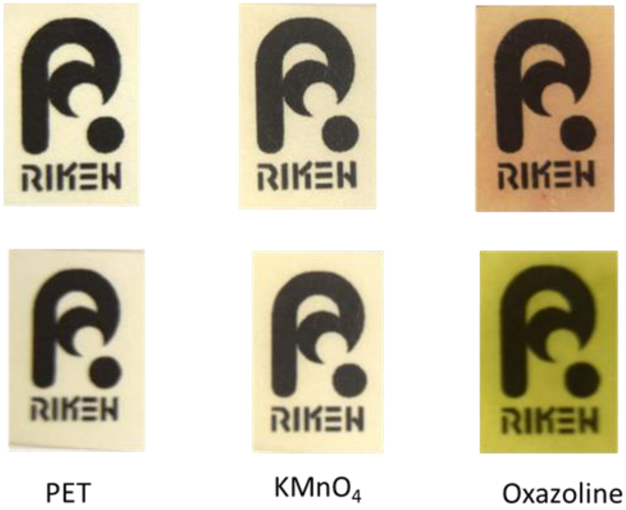

Figure S2. The grafting of TCPP was successful only for the PET films that were initially coated with oxazoline. While there was an observable change in color for pristine PET and $KMnO_4$-treated PET, the films do not transition to green upon exposure to Hg^{2+} ions except for the one containing oxazoline. (Top - before exposure to Hg^{2+}; Bottom - after exposure to Hg^{2+}, Adapted with permission to use the logo copyrighted by RIKEN.)

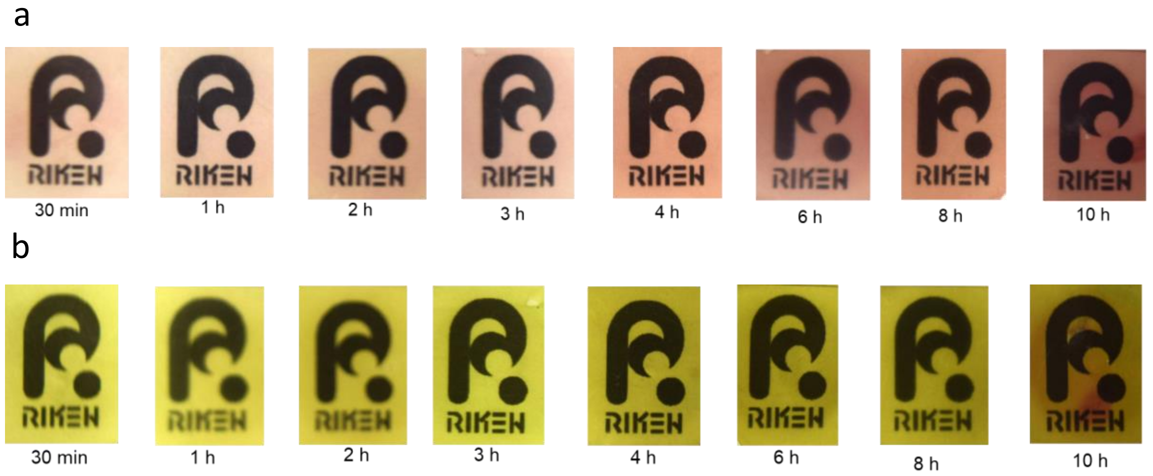

Figure S3. TCPP grafting on oxazoline-coated PET at different times at 110°C. (a) TCPP-grafted films before exposure to Hg^{2+} and (b) after exposure to Hg^{2+}. The longer the exposure, the more reddish the films become. Also, 30 min of grafting can create a film that can already transition from red to green. The films produced after prolonged grafting took longer time before completely transitioning to green when treated with Hg^{2+} solution. This can be attributed to the higher number of grafted TCPP moieties on the film that must be metalated first before complete green coloration can be observed. For the purpose of this study, TCPP films grafted for 3 h were utilized for the rest of the study. (Adapted with permission to use the logo copyrighted by RIKEN.)

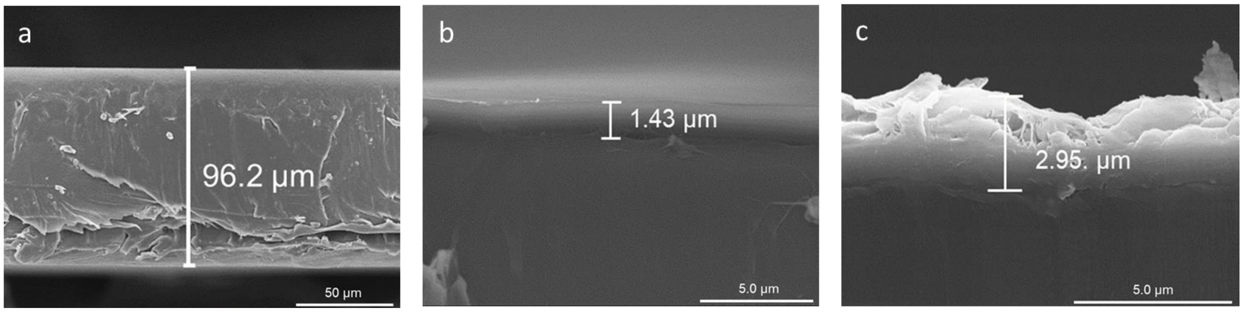

Figure S4. Thickness measurements by SEM for (a) -COOH-rich PET, (b) spin-coated oxazoline and (c) oxazoline-TCPP layer

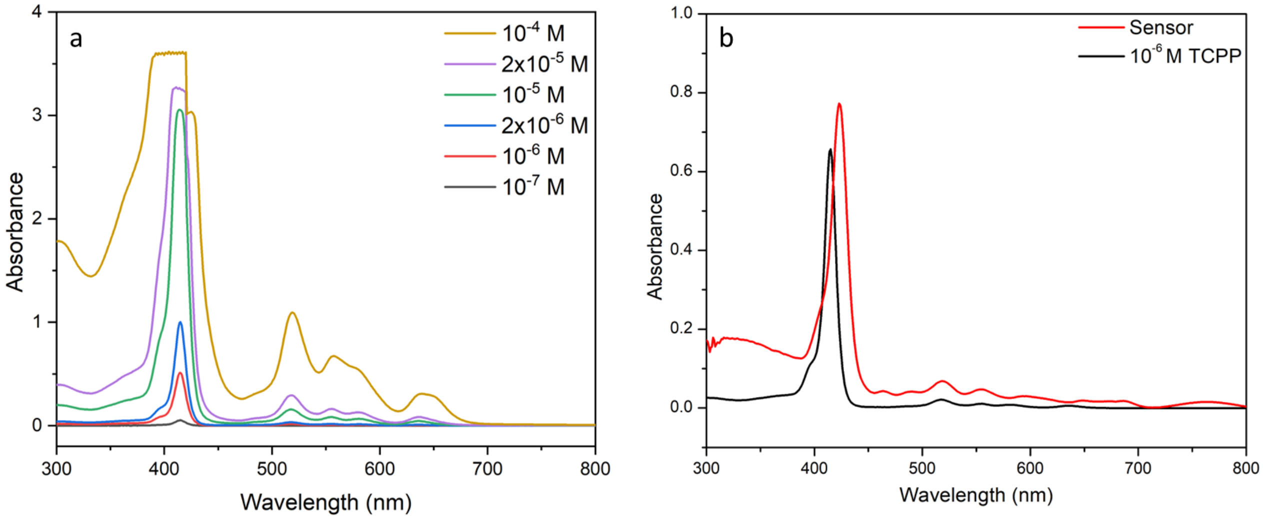

Figure S5. (a) UV-Vis profiles of TCPP upon increasing concentration in 0.01 M NaOH solution and (b) Comparison of the UV-Vis profiles of 10^{-6} M TCPP and the fabricated probe.

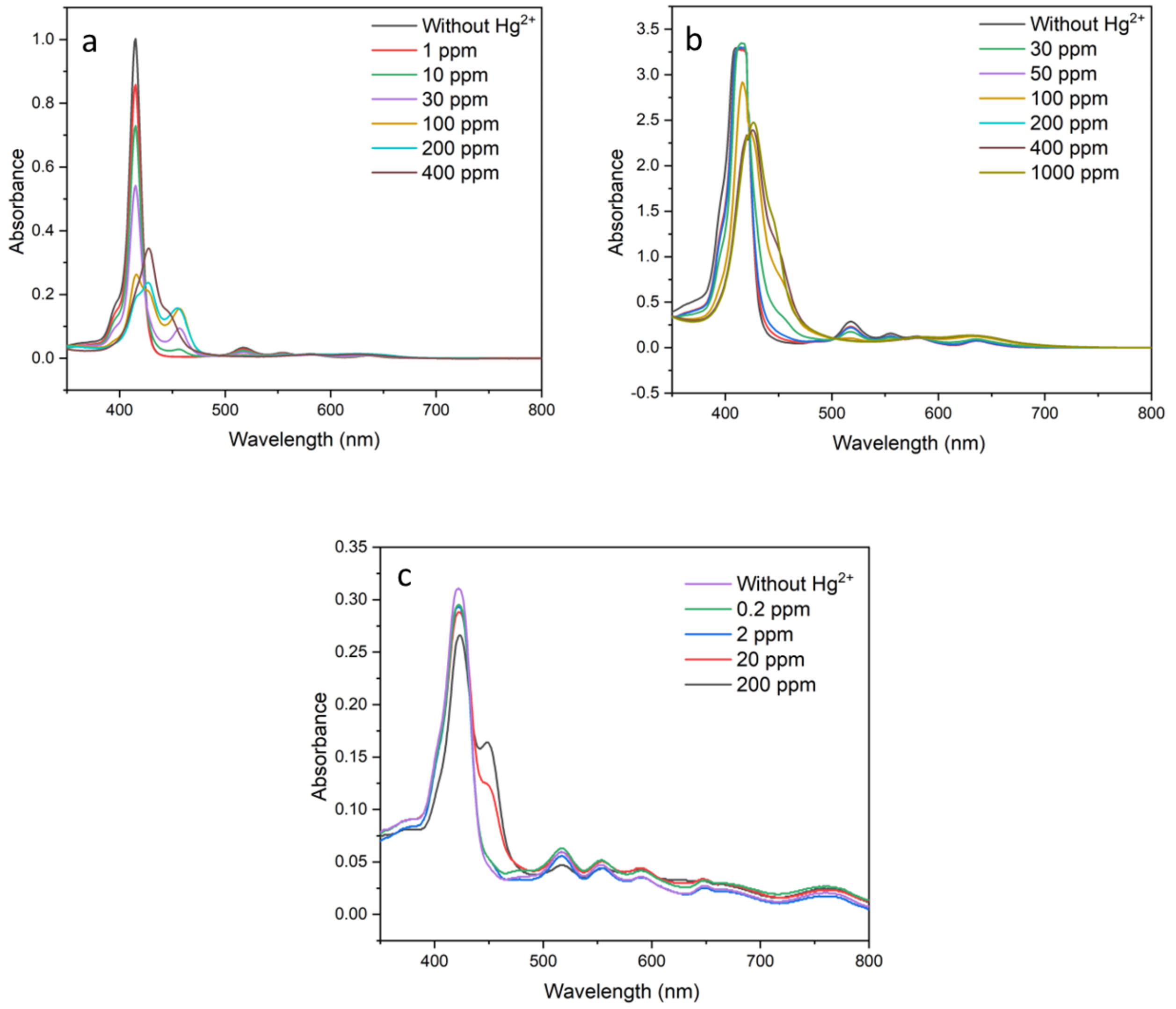

Figure S6. UV-Vis profiles of (a) 2 μM TCPP, (b) 25 μM TCPP solution and (c) the fabricated film upon exposure to increasing concentrations of Hg^{2+}.

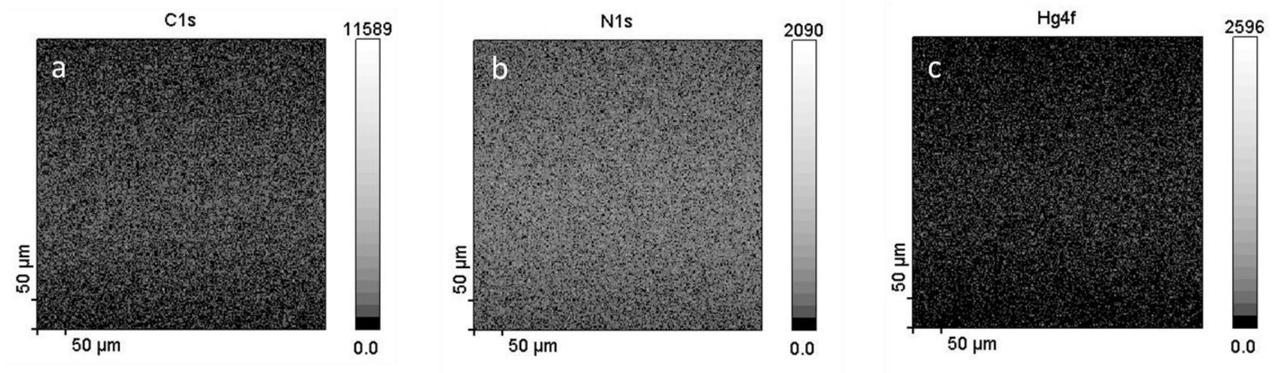

Figure S7. XPS images of the atomic percent distribution of core level electrons for (a) C 1s, (b) N 1s and (c) Hg 4f on the surface of the metal-bound colorimetric probe.

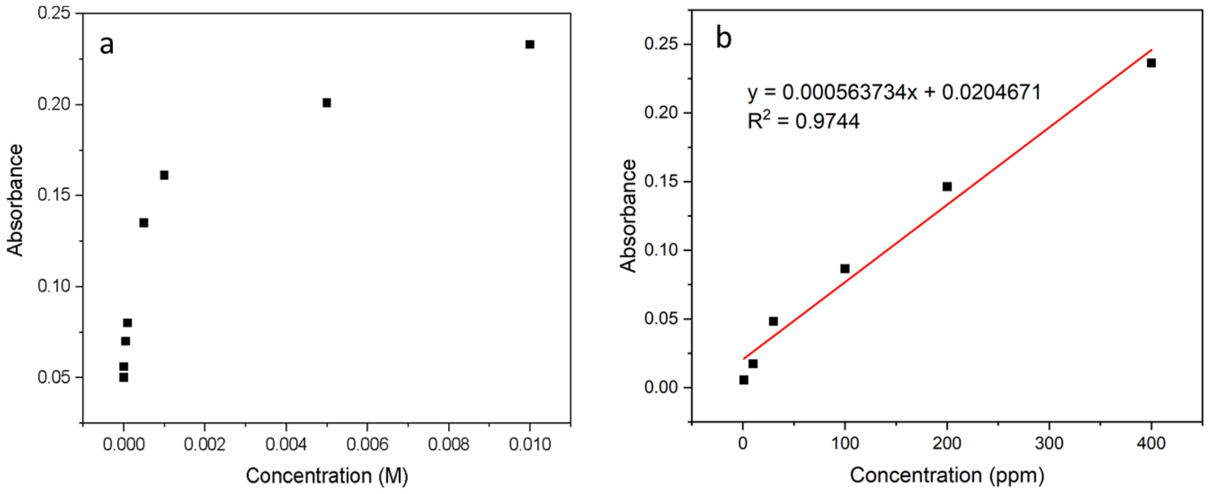

Figure S8. (a) Plot of absorbance against concentration using different standard solutions and (b) calibration curve for Hg^{2+} analysis

Table S2. Comparison of the fabricated colorimetric probe's performance with other Hg^{2+} sensors/probes previously reported

Material Description	Quantification Method / Limit of Detection (ppb)	Linear Range	Analysis time	pH	Reusability Studies
Fluorescent Copper Nanoclusters on Egg Shell Membrane with L-Cys as Reducing Agent [1]	Visualization under UV: 1.1 µM (220 ppb)<br><br>Fluorescence: 0.52 µM (104 ppb)	5 – 400 µM	1 h (naked eye detection)<br><br>30 min (fluorescence)	Not reusable	None
Fluorescent CdTe-Quantum-Dots-Modified Silica Nanospheres on Paper [2]	Smartphone camera: 2.83 ppb<br><br>Fluorescence: 0.38 ppb	5 – 100 ppb (smart phone)<br><br>1 – 100 ppb (fluorescence)	90 s	7.5	None
Polymer- Grafted TiO_2 Nanoparticles [3]	Naked eye: 10 ppb	No linear range provided.<br><br>Only for qualitative detection.	30 s	8 (not suitable for acidic conditions)	4 times
Cysteine-Capped Ag Nanoparticles [4]	UV-Vis: 45 nM (9 ppb)<br><br>Naked eye: 2.5 µM (501 ppb)	1 - 2500 nM (UV-Vis)	5 min	7	None
Green Synthesized Ag Nanoparticles [5]	UV-Vis: 2.48 µM (497 ppb)<br><br>DPV: 1.46 µM (293 ppb)<br><br>Naked eye: 45 µM (9,027 ppb)	5 – 45 µM (UV-Vis)<br><br>5 – 45 µM (DPV)	Not indicated	6 (Higher and lower pH prevent optimal sensing)	None
Coumarin-based Fluorescent Probe on Paper Strip [6]	Fluorescence: 8.3 nM (1.66 ppb)<br><br>Visualization under UV: 0.2 µM (40 ppb)	Not specified	6 min	7.2	None
Hg Ion-Imprinted Polymer on QCM Gold Chips [7]	Quartz Crystal Microbalance: 14.17 ppb	42.94 ppb to 2 ppm	Not specified but analysis QCM analysis times take longer periods to finish.	3.77	6 times
Blue/Red Carbon Dots (CDs) Ratiometric Fluorescent Sensors [8]	Fluorescence: 0.028 µM (5.61 ppb)	0.05 – 7.0 µM	2 min incubation	7	None
Poly Allyl Amine Hydrochloride-Capped AgNPs [9]	UV-Vis: 1 nM (0.2 ppb)	0.001 to 2.5 µM	5 min incubation and 2.5 min detection process	5	None

NMP-Stabilized AuNPs [10]	UV-Vis :0.3 µM (60.2 ppb)<br><br>Naked eye: 15 µM (3,008 ppb)	1 – 30 µM	10 min incubation	4 - 7	None
Vancomycin-Functionalized & Polyethyleneimine Stabilized Au Nanoparticles [11]	Fluorescence: 0.998 nM (0.2 ppb)	2 – 64 µM	Not specified	7.0	None
Ru@UiO-66-NH2 Composite [12]	Fluorescence: 0.053 µM (10 ppb)	0.1 – 50 µM	10 min incubation	7.0	None
1,6-Hexanedithiol-Modified Au/Ag SPR Chip [13]	SPR: 0.4 µM (80.2 ppb)	0 – 498 µM	4 min	Not specified	None
Ca-MOF [14]	ASV: 0.6 ppb	2 - 40 ppb	6 min	3 - 8	2 months
Porphyrin -Quinoline Dyad (Homogeneous) [15]	Fluorescence: 22 nM (4.41 ppb)	0.3 - 20 µM	Fast	5 - 9	None
Amphiphilic Porphyrin on Glass [16]	Fluorescence: 1 µM (200 ppb)	1 - 100 µM	Not specified	5 - 9	6 times
Porphyrin-Functionalized Au@SiO_2 Nanoparticles [17]	Fluorescence: 1.2 ppb	5 - 20 ppb	Not Specified	4 - 10	None
Porphyrin-Functionalized Polyacrylonitrile Fiber [18]	Naked eye: 20 ppb	Only qualitative detection	Concentration-dependent	3 - 11	50
Porphyrin-Functionalized Fe_3O_4@SiO_2 [19]	40 µ M (8,023 ppb)	10^{-5} to 10^{-4} M	1 min	7	Reusable
Porphyrin-based Langmuir Films on Polyvinyl Chloride [20]	Fluorescence: 2 ppb	10^{-5} – 10^{-8} M	15 min	Not specified	5 times
Porphyrin Derivative on Colour Catcher on Fabric [21]	Fluorescence: 10 nM (2 ppb)	10^{-8} – 10^{-4} M	15 min	7	None
PMMA films doped with Benzoporphyrin and Porpyrin-2-yl-pyridine [22]	Fluorescence: 0.16 µM (32.1 ppb)	10^{-4} to 10^{-6} M	Not indicated	7	None
Porphyrin in Highly Crosslinked Polymer [23]	Fluorescence:10 ppb	20 – 70 ppb	30 s	Neutral	None
Porphyrin grafted on sol-gel matrix [24]	Fluorescence: 10^{-3} M (2 x 10^5 ppb)	Not indicated	30 min	4	None
This Work	Naked eye detection: 10^{-7} M (20 ppb)<br><br>UV-Vis: 10^{-8} M (2 ppb)	0.739 to 295.5 ppm	Naked eye: Dependent on concentration<br><br>UV-Vis: 10 min	2 - 13	At least 50 times

References

[1] C. Zhang, M. Liang, C. Shao, Z. Li, X. Cao, Y. Wang, Y. Wu, S. Lu, Visual Detection and Sensing of Mercury Ions and Glutathione Using Fluorescent Copper Nanoclusters, ACS Appl. Bio Mater. 6 (2023) 1283–1293. https://doi.org/10.1021/acsabm.3c00031.

[2] J. Han, H. Liu, J. Qi, J. Xiang, L. Fu, X. Sun, L. Wang, X. Wang, B. Li, L. Chen, A Simple and Effective Visual Fluorescent Sensing Paper-Based Chip for the Ultrasensitive Detection of Mercury Ions in Environmental Water, Sensors. 23 (2023). https://doi.org/10.3390/s23063094.

[3] B. Heidari, P. Zarshenas, R. Sedghi, M.R. Nabid, R.S. Varma, Highly selective and sensitive recognition of multi-ions in aqueous solution based on polymer-grafted nanoparticle as visual colorimetric sensor, Sci. Rep. 14 (2024) 1–14. https://doi.org/10.1038/s41598-023-50627-x.

[4] V.R. Samuel, K.J. Rao, A rapid colorimetric dual sensor for the detection of mercury and lead ions in water using cysteine capped silver nanoparticles, Chem. Phys. Impact. 6 (2023) 100161. https://doi.org/10.1016/j.chphi.2023.100161.

[5] M.S. Punnoose, D. Bijimol, T. Abraham, N.J. Plathanam, B. Mathew, Green Synthesized Unmodified Silver Nanoparticles as Reproducible Dual Sensor for Mercuric Ions and Catalyst to Abate Environmental Pollutants, Bionanoscience. 11 (2021) 739–754. https://doi.org/10.1007/s12668-021-00883-w.

[6] S. Muthusamy, K. Rajalakshmi, D. Zhu, W. Zhu, S. Wang, K.B. Lee, H. Xu, L. Zhao, Dual detection of mercury (II) and lead (II) ions using a facile coumarin-based fluorescent probe via excited state intramolecular proton transfer and photo-induced electron transfer processes, Sensors Actuators B Chem. 346 (2021) 130534.

https://doi.org/10.1016/j.snb.2021.130534.

[7] K.M. Low, X. Lin, H. Wu, S.F.Y. Li, Ion-Imprinted Polymer-Based Sensor for the Detection of Mercury Ions, Polymers (Basel). 16 (2024) 652. https://doi.org/10.3390/polym16050652.

[8] Y. Liu, L. Chen, X. Su, L. Wang, Y. Jiao, P. Zhou, B. Li, R. Duan, G. Zhu, Constructing an eco-friendly and ratiometric fluorescent sensor for highly efficient detection of mercury ion in environmental samples, Environ. Sci. Pollut. Res. Int. 31 (2024) 4318–4329. https://doi.org/10.1007/s11356-023-31167-3.

[9] V.R. Samuel, K.J. Rao, A colorimetric sensor for the stable and selective detection of mercury ions using PAH-capped silver nanoparticles in an aqueous medium, Appl. Nanosci. 14 (2024) 33–42. https://doi.org/10.1007/s13204-023-02948-6.

[10] X. Shao, D. Yang, M. Wang, Q. Yue, A colorimetric detection of Hg2+ based on gold nanoparticles synthesized oxidized N-methylpyrrolidone as a reducing agent, Sci. Rep. 13 (2023) 1–8. https://doi.org/10.1038/s41598-023-49551-x.

[11] A.K. Tiwari, H.P. Yadav, M.K. Gupta, R.J. Narayan, P.C. Pandey, Synthesis of vancomycin functionalized fluorescent gold nanoparticles and selective sensing of mercury (II), Front. Chem. 11 (2023) 1–10. https://doi.org/10.3389/fchem.2023.1238631.

[12] P. Jia, K. Yang, J. Hou, Y. Cao, X. Wang, L. Wang, Ingenious dual-emitting Ru@UiO-66-NH2 composite as ratiometric fluorescence sensor for detection of mercury in aqueous, J. Hazard. Mater. 408 (2021) 124469. https://doi.org/10.1016/j.jhazmat.2020.124469.

[13] G. Jiang, Y. Miao, J. Wang, H. Shao, H. Chen, P. Tao, W. Wang, Q. Yu, W. Peng, X. Zhou, Specific detection of mercury ions based on surface plasmon resonance sensor modified with 1, 6-hexanedithiol, Sensors Actuators A Phys. 356 (2023).

https://doi.org/10.1016/j.sna.2023.114343.

[14] C. Kokkinos, A. Economou, A. Pournara, M. Manos, I. Spanopoulos, M. Kanatzidis, T. Tziotzi, V. Petkov, A. Margariti, P. Oikonomopoulos, G.S. Papaefstathiou, 3D-printed lab-in-a-syringe voltammetric cell based on a working electrode modified with a highly efficient Ca-MOF sorbent for the determination of Hg(II), Sensors Actuators, B Chem. 321 (2020) 128508. https://doi.org/10.1016/j.snb.2020.128508.

[15] Z.X. Han, H.Y. Luo, X.B. Zhang, R.M. Kong, G.L. Shen, R.Q. Yu, A ratiometric chemosensor for fluorescent determination of Hg2+ based on a new porphyrin-quinoline dyad, Spectrochim. Acta - Part A Mol. Biomol. Spectrosc. 72 (2009) 1084–1088. https://doi.org/10.1016/j.saa.2009.01.003.

[16] L.S. Dolci, E. Marzocchi, M. Montalti, L. Prodi, D. Monti, C. Di Natale, A. D'Amico, R. Paolesse, Amphiphilic porphyrin film on glass as a simple and selective solid-state chemosensor for aqueous Hg2+, Biosens. Bioelectron. 22 (2006) 399–404. https://doi.org/10.1016/j.bios.2006.05.013.

[17] Y. Cho, S.S. Lee, J.H. Jung, Recyclable fluorimetric and colorimetric mercury-specific sensor using porphyrin-functionalized Au@SiO2 core/shell nanoparticles, Analyst. 135 (2010) 1551–1555. https://doi.org/10.1039/c0an00137f.

[18] X. Liu, X. Liu, M. Tao, W. Zhang, A highly selective and sensitive recyclable colorimetric Hg2+ sensor based on the porphyrin-functionalized polyacrylonitrile fiber, J. Mater. Chem. A. 3 (2015) 13254–13262. https://doi.org/10.1039/c5ta02491a.

[19] L. Sun, Y. Li, M. Sun, H. Wang, S. Xu, C. Zhang, Q. Yang, Porphyrin-functionalized Fe3O4@SiO2 core/shell magnetic colorimetric material for detection, adsorption and removal of Hg2+ in aqueous solution, New J. Chem. 35 (2011) 2697–2704.

https://doi.org/10.1039/c1nj20307j.

[20] E. V. Ermakova, E.O. Koroleva, A. V. Shokurov, V. V. Arslanov, A. Bessmertnykh-Lemeune, Ultra-thin film sensors based on porphyrin-5-ylphosphonate diesters for selective and sensitive dual-channel optical detection of mercury(II) ions, Dye. Pigment. 186 (2021). https://doi.org/10.1016/j.dyepig.2020.108967.

[21] F. Caroleo, G. Magna, C. Damiano, M. Cavalleri, E. Gallo, C. Di Natale, R. Paolesse, Colour Catcher® sheet beyond the laundry: A low-cost support for realizing porphyrin-based mercury ion sensors, Sensors Actuators B Chem. 364 (2022) 2–9. https://doi.org/10.1016/j.snb.2022.131900.

[22] N.M.M. Moura, C. Nuñez, S.M. Santos, M.A.F. Faustino, J.A.S. Cavaleiro, M.G.P.M.S. Neves, J.L. Capelo, C. Lodeiro, Functionalized porphyrins as red fluorescent probes for metal cations: Spectroscopic, MALDI-TOF spectrometry, and doped-polymer studies, Chempluschem. 78 (2013) 1230–1243. https://doi.org/10.1002/cplu.201300216.

[23] Y. Hu, L. Meng, Q. Lu, “Fastening” porphyrin in highly cross-linked polyphosphazene hybrid nanoparticles: Powerful red fluorescent probe for detecting mercury ion, Langmuir. 30 (2014) 4458–4464. https://doi.org/10.1021/la500270t.

[24] D. Delmarre, R. Méallet, C. Bied-Charreton, R.B. Pansu, Heavy metal ions detection in solution, in sol-gel and with grafted porphyrin monolayers, J. Photochem. Photobiol. A Chem. 124 (1999) 23–28. https://doi.org/10.1016/S1010-6030(99)00046-5.
